# Interferon lambda in respiratory viral infection: immunomodulatory functions and antiviral effects in epithelium

**DOI:** 10.3389/fimmu.2024.1338096

**Published:** 2024-03-01

**Authors:** Yong-Guang Liu, Su-Wei Jin, Shan-Shan Zhang, Tian-Ji Xia, Yong-Hong Liao, Rui-Le Pan, Ming-Zhu Yan, Qi Chang

**Affiliations:** Institute of Medicinal Plant Development, Chinese Academy of Medical Sciences and Peking Union Medical College, Beijing, China

**Keywords:** interferon lambda, respiratory tract, viral infection, type III interferon, immunity, inflammation

## Abstract

Type III interferon (IFN-λ), a new member of the IFN family, was initially considered to possess antiviral functions similar to those of type I interferon, both of which are induced via the JAK/STAT pathway. Nevertheless, recent findings demonstrated that IFN-λ exerts a nonredundant antiviral function at the mucosal surface, preferentially produced in epithelial cells in contrast to type I interferon, and its function cannot be replaced by type I interferon. This review summarizes recent studies showing that IFN-λ inhibits the spread of viruses from the cell surface to the body. Further studies have found that the role of IFN-λ is not only limited to the abovementioned functions, but it can also can exert direct and/or indirect effects on immune cells in virus-induced inflammation. This review focuses on the antiviral activity of IFN-λ in the mucosal epithelial cells and its action on immune cells and summarizes the pathways by which IFN-λ exerts its action and differentiates it from other interferons in terms of mechanism. Finally, we conclude that IFN-λ is a potent epidermal antiviral factor that enhances the respiratory mucosal immune response and has excellent therapeutic potential in combating respiratory viral infections.

## 1 Introduction

Respiratory infection is a long-term and severe threat to human health. According to the WHO Global Health Estimates 2019 report, respiratory infections including pneumonia, resulted in the fourth highest number of deaths worldwide from 2000 to 2019. Viruses are the most common cause of respiratory infections. An outpatient survey revealed that viral infections accounted for 12.1% of cases, viral coinfections accounted for 44.2% of cases, and both viral and bacterial coinfections accounted for 10% of cases (1). The respiratory infections caused by the SARS coronavirus, avian influenza A virus (H5N1), influenza A virus (H1N1) in 2009, and the new coronavirus (SARS-CoV-2) in 2019 have demonstrated that viruses are important causative agents of severe pneumonia (2).

According to the Johns Hopkins University COVID-19 dynamic tracking data, as of March 2023, more than 670 million people have been infected with SARS-CoV-2 worldwide, and the number of related deaths is as high as 6.875 million (https://coronavirus.jhu.edu/). The most common complication of COVID-19 is pneumonia, which initially presents with cough and shortness of breath and progresses to acute respiratory distress syndrome within a week in severe patients (3).

Interferons (IFNs) are a class of cytokines that respond when stimulated by infection or inflammation. IFNs can be divided into three types, viz., type I IFN with more than 20 subtypes (including IFN-α, IFN-β, IFN-ε, IFN-κ, and IFN-ω), type II IFN (IFN-γ), and type III IFN (IFN-λ, including four subtypes, IFN-λ1–4.). IFN-λ1 (IL-29), IFN-λ2 (IL-28A), and IFN-λ3 (IL-28B) were identified in 2002/2003 by two independent research groups (4, 5). IFN-λ4 was identified in 2013 based on RNA sequence data from Poly (I: C)-treated primary hepatocytes (6). Unlike that in humans, mice have only two genes, viz., *Ifnl2* and *Ifnl3*, encoding functional IFN-λ. *Ifnl1* is a pseudogene, and the genomic region encoding IFN-λ4 has not been reported (7–9).

IFN-λ, a relatively young member of the interferon family, is considered to play a nonredundant role in resistance to viral infections and in innate immunity at the epithelial barrier surface. Previous clinical trials have demonstrated good antiviral ability of IFN-λ (10). A double-blind, placebo-controlled trial showed that IFN-λ accelerated viral clearance in patients with COVID-19 and was more likely to be undetectable on day 7 compared with that in the placebo group, demonstrating that IFN-λ has a good potential to shorten the duration of viral shedding and for the treatment of COVID-19 (11). A study in mice lacking the functional receptor of IFN-λ showed that IFN-λ plays a vital role in resistance to several human pathogens that infect the respiratory tract, such as H1N1, respiratory syncytial virus (RSV), human metapneumovirus, and SARS (12). Nevertheless, there has been relatively limited research on IFN-λ because of the limitation of its expression in nonepithelial cells in body tissues. In addition to its direct antiviral effect (innate immune), IFN-λ triggers an antigen-specific immune response through the activation and regulation of T and B cells, suggesting its unique ability to bridge the gap between innate and adaptive immune response (13). IFN-λ is functionally similar to type I IFN in that both induce the transcription of IFN-stimulated genes (ISGs) via the Janus kinase (JAK)–signal transducer and activator of transcription (STAT) pathway; however, the differences in their expression sites are more worthy of consideration.

In this review, we summarize the research progress in past 20 years on the role of IFN-λ in regulating the epithelial cell barrier and cell immunity in virus-induced respiratory inflammation, the role of intracellular sensors that recognize virus signals and induce IFN-λ production, and the induction mechanism of IFN-λ, and demonstrate the application of IFN-λ in viral pneumonia and how this information can be applied to future therapeutic strategies.

## 2 IFN-λ targeting the immune regulation of epithelial cell barriers

**Figure 1:**
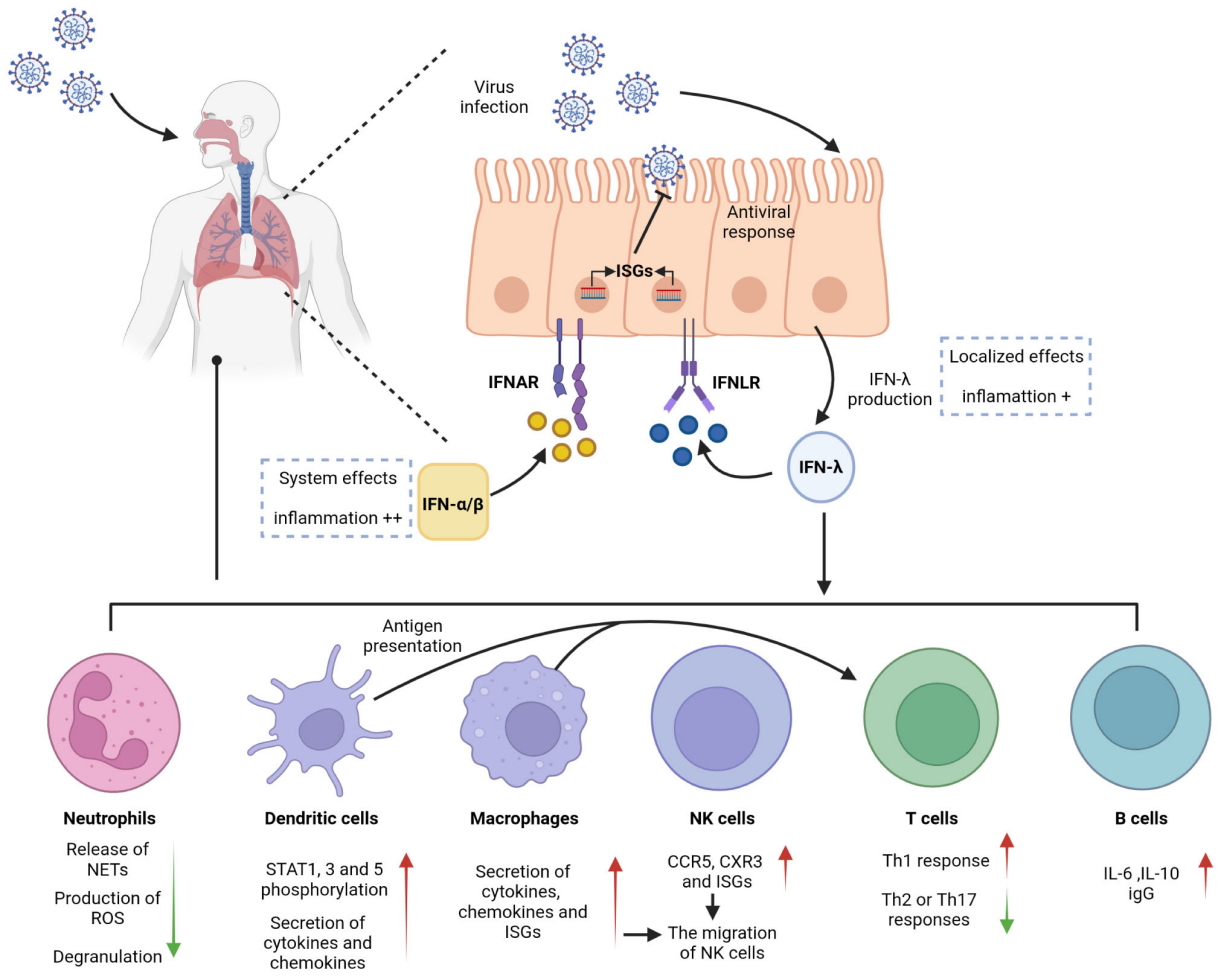
Antiviral effects of IFN-λ on barrier surfaces and immune cells. IFN-λ is an interferon produced in response to viral infection. Respiratory epithelial cells can respond to both IFN-λ and IFN-α/β, activating antiviral responses. IFN-λ signals through a receptor complex, IFNLR, consisting of IFNLR1 and IL10R2. IFNLR activates and induces the expression of ISGs, recruits and activates immune cells, and exerts an antiviral effect. IFNLR expression is limited to epithelial cells and subsets of immune cells, so IFN-λ exerts a localized antiviral effect in the early stage of respiratory inflammation, which can reduce the side effects and inflammation caused by the systemic effects of IFN-α/β.

The cellular barrier of the innate immune system provides a robust defense mechanism against viral invasion. Pattern recognition receptors (PRRs) on the cell surface sense different pathogens and induce the production of immune mediators such as IFNs to provide protection for the organism. Viral infection primarily induces the production of type I and type III IFNs, which share critical functional similarities using the JAK/STAT pathway to induce the transcription of antiviral ISGs. IFN-λ is involved in controlling viral transmission, particularly in reducing viral transmission from the upper respiratory tract to the lungs (14). IFN-λ binds to a heterodimeric receptor complex consisting of a unique α-chain IFN-λ receptor 1 (IFNLR1) and a β-chain IL-10 receptor (IL-10R2), and activates the STAT phosphorylation signaling cascade (15, 16). In contrast to the receptor of type I IFNs (IFNAR), which is expressed in almost all types of cells, the IFN-λ receptors IFNLR1 and IL-10R2 are widely distributed in epithelial cells, indicating that IFN-λ contributes to specific immune mechanisms of epithelial cells that protect organs (17–20). IFN-λ acts as a front-line defense against viruses, being earliest and predominantly produced at the epithelial cells during viral infection, inhibiting initial viral transmission without activating inflammation and providing early infection control at the barrier surface (**Figure 1**). This minimizes damaging inflammatory responses to the organism and avoids unnecessary frequent triggering of the type I IFN system, which can result in increased inflammation. Type I IFNs provide further antiviral action in the event of an insufficient local response provided by IFN-λ, but excessive reaction can cause excessive release of inflammatory cytokines, which results in the development of an inflammatory cytokine storm and further induction of severe lung infection (8, 21, 22). Early in viral infection, Ifnlr1-/- mice show higher viral loads in the lung, more viral particles, and higher inflammatory cell counts in BALF than wild-type mice, indicating that IFN-λ is critical for viral control in the early stage of infection (21, 23, 24). However, other studies have found that sustained activation of IFN-λ in the lung epithelial cells reduces lung barrier integrity and contributes to the accumulation of immune cells. Pathogens are more likely to enter the airways, which correspondingly predisposes the host to fatal secondary infections, when epithelial cells are chronically and continuously exposed to IFN-λ (25, 26). This also suggests that the duration of IFN-λ treatment must be determined on a case-by-case basis when IFN-λ is used as a drug to treat viral infection.

## 3 IFN-λ targeting the regulation of cell immunity

### 3.1 Neutrophils

Neutrophils, produced in large numbers in the bone marrow through progressive differentiation of myeloid progenitor cells (CMP), are recognized as essential effector cells of the innate immune system. As the most abundant type of leukocyte in the circulation, neutrophils are front-line troopers and primary immune cells in the acute inflammatory response (27–29) and are first recruited to the sites of inflammation following pathogen invasion or tissue injury. During this time, inflammatory mediators produced by cells in inflamed tissues bind to G protein-coupled receptors and activate neutrophils. Neutrophils can also be directly activated by pathogen-associated molecular patterns (PAMPs) and/or damage-associated molecular patterns (DAMPs). In particular, in mice infected with influenza A virus, neutrophils are the primary responsive immune cells to inflammation with the highest level of IFNLR1 expression, and IFN-λs induce neutrophils to produce large amounts of ISGs that inhibit viral invasion by degrading viral RNA. This effect not only inhibits viral translation and replication but also limits viral spread to neighboring cells (21, 30). Neutrophil functional responses are diverse, including phagocytosis, production of reactive oxygen species (ROS), and release of neutrophil extracellular traps (NETs) with net structures, which are emerging therapeutic targets for inflammation. The production of ROS and NETs results in cytotoxicity but does not affect the phagocytic function or cytokine production of neutrophils (26, 31–33). An *in vitro* experiment demonstrated IFN-λ1 modulated neutrophil function in a threshold manner and inhibited the release of NETs by regulating autophagy to exert an anti-inflammatory effect (34). Several studies have demonstrated that IFN-λ receptors are expressed in both mouse and human neutrophils and that the expression of IFNLR1 in human neutrophils can be improved when stimulated by LPS, an activator of inflammatory cytokines (35, 36). Blazek et al. found that *in vitro* stimulation of neutrophils with IFN-λ can activate the JAK-STAT pathway and induce STAT1 phosphorylation. IFN-λ also regulates the tissue-damaging function of neutrophils in a nontranslational and nontranscriptional manner. When new protein synthesis is inhibited using cycloheximide and STAT expression is inhibited using fludarabine, IFN-λ still exerts its anti-inflammatory effects in neutrophils. Even without STAT, IFN-λ can regulate AKT activation by activating JAK to induce JAK phosphorylation and reduce neutrophil ROS production and degranulation (**Figure 1**) (36). The regulation of neutrophil function by IFN-λ is influenced by the tissue microenvironment, which is a chronic inflammatory environment with massive infiltration of inflammation-related cells and associated inflammatory cytokines at the site of injury.

### 3.2 Dendritic cells

Dendritic cells (DCs) are a highly heterogeneous population of cells and can be divided into three major subsets, viz., plasmacytoid DCs (pDCs), originating from both myeloid DC progenitor and lymphoid progenitors, and conventional DC1 and DC2 subsets (cDC1 and cDC2), originating from myeloid progenitors. DCs are typically distributed in the bone marrow and lymph, and less presented in the skin or mucous membrane of the respiratory tract and lung. Despite low expression levels of DCs in organs, they play a vital role in linking innate and adaptive immunity in the immune response (37–39). The ability to cross-present antigens is considered a characteristic of DCs and makes their responses to IFN-λ highly significant (40). Several studies have identified pDCs as the major IFN-λ-responsive cells in peripheral blood. Analysis of human peripheral blood leukocytes from healthy donors revealed that pDCs expressed high levels of IFN-λ receptors, and IFN-λ1 was observed to upregulate STAT1, STAT3, and STAT5 phosphorylation responses in pDCs in a time- and dose-dependent manner (**Figure 1**) (41, 42).

Different subsets of DCs are recruited to the nasal mucosa and respiratory tract during respiratory infections by viruses (37, 43) such as respiratory syncytial virus (RSV), influenza virus, and parainfluenza virus (44). IFN-λ expression varies among DC subsets, and mouse CD8α^+^ DCs have been demonstrated to be the most effective in antigen presentation among all DC subpopulations, although human cDC1 has better exogenous antigen presentation and produces more adult IFN-λ (38, 40, 45). After induction with Poly (I: C), mouse CD8α^+^ DCs and human counterparts BDCA3^+^ DCs highly expressed Toll-like receptor 3 (TLR3) and cross-presented antigens and were induced to produce large amounts of IFN-λ (46); however, induction with the the TLR4 activator LPS did not increase IFN-λ expression (47). Analysis of the expression of IFN-λ on immune cells from virus-responsive patients revealed that both the receptors IFNLR1 and IL-10R2 were significantly expressed in DCs. pDCs can also combinatorially express TLR-7 and interferon regulatory factor 7 (IRF-7), whereas IFN-λ is a TLR-7 activation product. Activated pDCs can produce immunomodulators such as IFN-λ, TNF-α and IL-6 to exert their anti-inflammatory effects (48). Immunization of mice with IL-28A-treated DCs promoted Th1 differentiation and inhibited Th2 or Th17 responses in their bodies, and the key to their anti-inflammatory effects was considered DC-mediated conversion of Th2 or Th17 to Th1 (49). DCs can produce IFN through multiple pathways, the most important of which is the antigen presentation function of DCs.

### 3.3 Macrophages

Macrophages are cells capable of recognizing, engulfing, and degrading cell debris and pathogens. Together with neutrophils, they are the first to respond at the beginning of inflammation by synthesizing and releasing inflammatory mediators (50). In coronavirus infection, macrophages play an antigen-presenting role and induce the production of type I and type III IFNs (51). Although CD14^+^ monocytes, precursors of macrophages, do not express IFN-λ receptors, their IFN-λ sensitivity is increased after differentiation into macrophages *in vitro* (52). TLR3 is a key receptor for the activation of IFN involved in antiviral responses, and IFN expression is increased in macrophages through the activation of the TLR3-JAK/STAT signaling pathway under the action of the TLR3 agonist Poly (I: C). The TLR3 pathway is activated by the virus, and the activation of JAK induces IRF3 and IRF7 phosphorylation, regulates IFN transcription, and increases ISG expression (53). A previous study showed that IFN-λ in rhinovirus-infected patients induced CXC chemokine mRNA expression in human peripheral blood monocytes, and the degree of mRNA expression positively correlated with the absolute and relative number of macrophages; however, the specific effect of IFN-λ on macrophages was not further revealed (54). Subsequently, further studies demonstrated that monocytes enter tissues and differentiate into macrophages, increasing the sensitivity to IFN-λ by inducing the expression of the IFN-λ receptor IFNLR1, which in turn amplifies the intensity and duration of the inflammatory cascade. Macrophages regulate the immune response to IFN-λ produced by the TLR stimulation of pathogens such as viruses. The effect of IFN-λ on macrophages can be divided into direct and indirect (**Figure 1**). Direct effects include increased cytotoxicity and phagocytosis of macrophages, enhanced recognition and presentation of antigens, and a corresponding increase in the secretion of cytokines (TNF and IL-1β) and chemokines that target inflammation. Indirect effects include the induction of CCR5 and CXR3 chemokine secretion by macrophages by IFN-λ, targeted recruitment of other immune cells such as Natural killer (NK) and T cells to the site of inflammation, and stimulation of increased secretion of ISGs to induce secretion and degranulation of other types of IFNs (52). The above-described evidence suggests that macrophages are the immune drivers of IFN-λ-mediated antiviral and inflammatory conditions.

### 3.4 NK cells

NK cells are a type of innate cytotoxic lymphocytes that play a crucial role in immunity by killing virus-infected or tumor cells and secreting cytokines and chemokines. NK cells are a class of innate immune cells that link innate and adaptive immunity. They can activate T cells by acting as antigen presenters, and their function of secreting cytokines and exerting cytotoxic effects by inducing apoptosis is jointly involved in the regulation of both the immune response and various immune-mediated inflammatory diseases (55–57). Unlike the well-recognized role of IFN-α in initiating NK cells, there is limited research on the relationship between IFN-λ and NK cells. In 2015, Souza-Fonseca-Guimaraes et al. found that NK cells in mice express mRNA for IL-28R, a specific receptor for IFN-λ1. In mice with specific deletion of IL-28R in NK cells, IFN-λ could promote the antimetastatic activity of NK cells compared with that in wild-type mice. This was the first report to show that IFN-λ can directly modulate NK cell function *in vivo*, either when it acts alone or in combination with type I and type II interferons (58). Based on their study, it was further found that IFN-λ deficiency impaired NK cell function and that the regulatory effect of IFN-λ on NK cells was direct rather than indirect (59). A recent study on the antiviral infection effects of NK cells and IFN-λ in young mice and the *in vivo* activation of NK cells by IFN-λ in cancer models further showed that IFN-λ binding to pathogen pattern recognition receptors on the mucosal surface recruited and activated NK cells to exert antiviral infection and immune effects *in vivo* (60, 61). Read et al. explained this effect of IFN-λ on NK cells by stating that IFN-λ stimulation induced the secretion of CCR5 and CXR3 chemokines and ISGs, resulting in the migration of NK cells and providing the impetus for their subsequent cytotoxicity (**Figure 1**) (52). Although there are fewer direct studies of IFN-λ and NK cells in viral pneumonia, the above-described findings will also provide new directions for future research on IFN-λ in pulmonary viral immunity.

### 3.5 T cells

T cells, i.e., T lymphocytes play a key role in adaptive immunity in response to respiratory viral infections. Proliferation and increased migration of antigen-specific T cells to the lung are characteristic of this immune response. T cells are divided into CD4^+^ helper T lymphocytes (Th) and CD8^+^ cytotoxic T lymphocytes based on different functions (62). Effector CD4^+^ and CD8^+^ T cells significantly modulate lung viral load, and the chemokines CCR5 and CXCR3 assist the migration of CD8^+^ T cells into the lung, whereas CD4^+^ T cells may be influenced only by CXCR3 (63). According to studies in rodents, CD8^+^ T cells could suppress respiratory tract inflammation by reducing eosinophil accumulation and airway mucus production (64). Human CD8^+^ T cells express IFN-λ receptors, and CD4^+^ T cells can enhance the antiviral effect of CD8+ T cells and also induce B cells to produce immunoglobulin (lg) (19, 65). Both human naive and memory CD4^+^ T cells express IL28R mRNA (66). During viral infection, DCs present antigens and migrate to the lungs, with an initial transient systemic T-cell lymphopenia, followed by a CD8^+^ T-cell response that mediates late viral clearance (67). In addition to its antiviral effects, IFN-λ increases the proportion of CD8^+^ T cells in the spleen of inoculated mice (13). The lungs of rhinovirus (RV)-infected mice exhibited increased expression of CD4^+^ T cells and elevated IFN-λ (68). These data allow us to consider their connection.

A recent study demonstrated that IFN can accelerate viral clearance in elderly patients with COVID-19 by delaying T-cell immunity (69). However, highly purified CD4^+^ and CD8^+^ T cells neither significantly expressed IL-28R nor upregulated known IL-28R target genes. IFN-λ signaling to T cells is mediated by other immune cells such as DC cells and macrophages (**Figure 1**). The prevailing view is that IFN-λ1 does not directly affect CD4^+^ T cells, and studies have demonstrated that IFN-λ do not participate in T-cell proliferation nor affect their regulatory activity and that IFN-λ1 may act through other specific cell subsets such as DCs (49, 70, 71).

### 3.6 Th1 and Th2 cells

Initial T cells are in a state of hyporesponsiveness known as quiescence. Upon receipt of signals from T cell receptors and signals triggered by specific transcription factors, primitive CD4^+^ T cells transmit the signals to generate different T helper (Th) subpopulations, Th1 and Th2 subsets were the first Th cell subsets to be identified, which secrete specific cytokines to function in the immune response to various viral infections (62, 72, 73). Virus-specific Th1 and cytotoxic T cells are critical for controlling viral replication, whereas Th2 cytokines conduct significant growth signals to B cells (74).

The difference in the roles of Th1 and Th2 cells is primarily due to the different cytokines they secrete. Compared with Th2 cells, the hallmark cytokines produced by Th1 cells are IFN-γ, IL-2, and IL-12, although Th1 cells are also good TNF-α producers and possess a unique ability to secrete cytotoxins (65). Analysis of primary bronchial epithelial cells (PBECs) from patients with severe chronic obstructive pulmonary disease (COPD) revealed significantly elevated TNF-α and IFN-γ levels, reduced IL-4 and IL-13 levels, and elevated Th1-dominated cytokine responses (75, 76). Well-differentiated PBECs derived from patients with severe COPD after human rhinovirus (HRV) infection exhibited impaired mRNA expression of IFN-λ and reduced expression of ISG compared with that in healthy controls, and underexpression of IFN-λ may be a factor in the frequent exacerbation of inflammation in patients with COPD (76, 77). These studies concluded that IFN-λs are potent adjuvants of the Th1 immune response and can enhance the immune response to antigens in a Th1-biased manner, and IL-12 induced by DCS acted indirectly on T cells to promote IFN-λ-induced Th1 immune excursions (**Figure 1**) (13, 49, 52).

DCs recognize and present specific antigens to induce Th2 cells in the airways and lungs to produce the hallmark inflammatory cytokines IL-4, IL-5 and IL-13. IL-4 induces the differentiation of Th2 cells and IgE isotype conversion of B lymphocytes (78), IL-5 regulates the development of eosinophils (79), and IL-13 enhances class II expression and IgE class conversion of B cells (80). The Th2 response primarily inhibits the differentiation of T cells, and these inflammatory factors mediate the coordination of inflammatory cell recruitment in the early stages of inflammation and subsequent tissue repair (64). After infection with rhinovirus in immature mice, an increase in the number of lung immune cells with upregulation of IL-13 was detected, which induced airway inflammation and mucus chemotaxis, and IFN-λ expression in the lungs was maintained at a high level 7 days after infection, suggesting that IFN-λ is associated with virus-induced Th2 immunity in the lungs (68). Previous studies have demonstrated that IL-29 can downregulate Th2 cytokines, especially IL-13 (54, 81). Studies have also shown that IFN-λ1 reduces lung eosinophilia and inhibits the release of IL-4, IL-5, and IL-13 from T cells after treatment of mice with pneumonia with a recombinant adenovirus expressing human IFN-λ1, demonstrating its ability to attenuate the lung inflammatory response (82, 83). Currently, it is generally accepted that IFN-λ exerts no direct effect on Th cells. Another study reported a different view, suggesting that IFN-λ1 directly inhibits the production of Th2-associated cytokines. The mechanism of action is independent of DC-mediated responses to inhibit Th2 cell polarization, which not only directly inhibits IL-13 production by naive and memory CD4^+^ T cells but also inhibits the differentiation of central memory T cells into effector T cells by regulating the expression of IL-4R and the transcription factor GATA3, but this does not affect T-cell proliferation (66). Nevertheless, those studies generally used *in vitro* cell cultures, and no direct effects have been found in studies on mouse or human systems.

Th1/Th2 cytokines in the average human body are in a state of equilibrium, and their effects cancel each other out to some extent. An imbalance in Th1/Th2 cytokines in the airways of asthmatic patients after rhinovirus infection manifests with elevated levels of Th2 marker inflammatory factors and a positive correlation with the severity of airway inflammation (84). Previous studies show that IFN-λ1 (IL-29) reduces the mRNA levels of Th2 cytokines (IL-4, 5, 13) and the amount of cytokine-secreting proteins, shifting the Th1/Th2 balance toward Th1 and producing a significant cytotoxic T-cell response (47, 66, 81, 83). Furthermore, IFN-λ mediates pulmonary protection by regulating IL-12 secretion from lung DC cells and promoting Th1 responses to inhibit Th2 responses (49). When patients vaccinated with IL-28B are stimulated by H1N1 influenza virus, macrophages are induced to secrete IL-12 to promote Th1 and cytotoxic responses, and the secretion of Th1 cytokines inhibits the differentiation of Th2 cells and the secretion of Th2 cytokines, which affects the Th1/Th2 balance (85, 86). In conclusion, IFN-λ may exert a critical regulatory effect on the Th1/Th2 balance primarily by regulating antigen-presenting cells such as DCs and macrophages.

### 3.7 B cells

Human B cells express IFN-λ receptors and respond to IFN-λs (19, 41, 87); however, the activation of B cells by IFN-λ was not detected in mice (18, 88). This difference deserves to be considered in future studies of IFN-λ. De Groen et al. found that both human naive and memory B cells express receptors for IFN-λ. In contrast to IFN-α, which alone fails to activate B cells, IFN-λ1 can increase cytokine and B-cell immunoglobulin (lg) levels by mediating B-cell activation through the upregulation of TLR7 expression, which becomes more potent after receiving signals from T helper cells (89). IFN-λ4 increases the release of immunosuppressive IL-10 when B cells are costimulated with the TLR9 ligand (19). This demonstrates that IFN-λ plays an enhancing role in humoral immunity. Analysis of the B-cell transcriptome revealed that IFN-λ-induced B-cell genes were stably expressed and cell-type-specific. Furthermore, IFN-λ induced B-cells to differentiate into plasma cells, release cytokines such as IL-6 and IL-10, and produce antibodies by activating the mTORC1 pathway in the cell body (**Figure 1**) (90). In a mouse model of pulmonary influenza, memory B cells migrate to the lung via CXCR3 chemokines and reside there for a long time, activating and producing influenza-specific antibodies when there is an active inflammatory response (91, 92). The IL-28B allele promotes B-cell activation and virus-specific IgG production in response to H1N1 virus stimulation (85).

## 4 Intracellular sensors for receiving virus signals

### 4.1 PRR-mediated recognition of viruses

**Figure 2:**
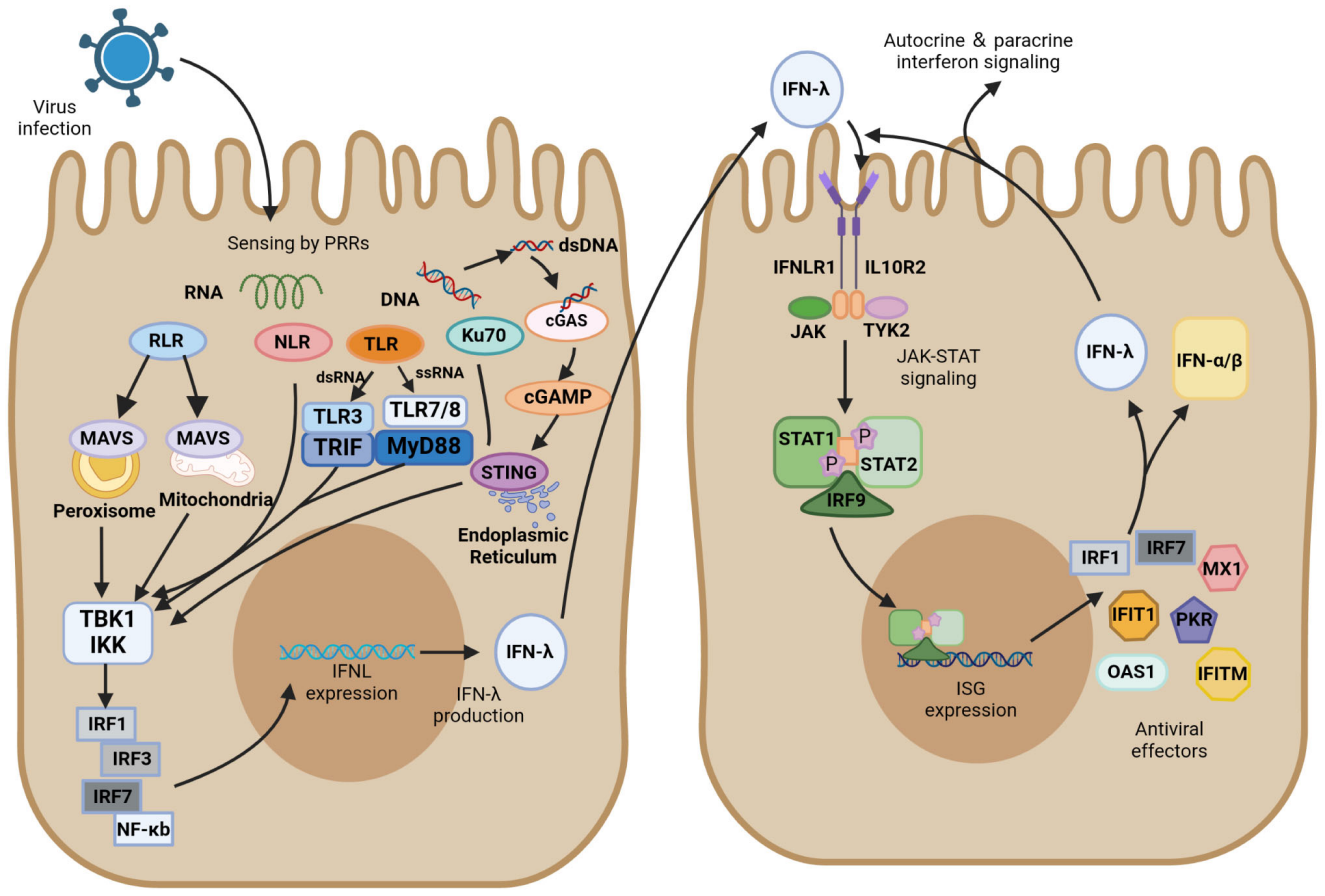
IFN-λ induction and antiviral signaling pathways. IFN-λ production is induced upon virus detection by pattern recognition receptors (PRRs), including Toll-like receptors (TLRs), RIG-I-like receptors (RLRs), and NOD-like receptors (NLRs), as well as the DNA sensor Ku70 and stimulator of interferon genes (STING). They further activate TANK-binding kinase 1 (TBK1) and the kinase IKK. These signals activate transcription factors of the interferon regulatory factor (IRF) family, which together with NF-κB promote IFN-λ expression. IFN-λ is transferred from infected cells to other cells in a paracrine form, signaling by binding to its heterodimeric receptor, IFNLR, which consists of IL28R1 and IL10R2 subunits. Upon binding, JAK and TYK2 kinases are activated, and they phosphorylate STAT1 and STAT2. JAK-STAT signaling induces the expression of IFN-stimulated genes (ISGs) and production of antiviral effectors, which act through various mechanisms to induce an antiviral state. In ISG induced by IFN-λ signaling, IRF1 and IRF7 encode the transcription factors IRF-1 and IRF-7, respectively, which promote IFN expression and IFN-λ positive feedback production of antiviral activity.

In respiratory virus infection, respiratory epithelial cells are the primary physical barrier to resist pathogen entry. After viral infection, PRRs in epithelial cells, which can recognize different PAMPs, immediately initiate the innate immune response. Most viruses infecting the respiratory tract, except herpesviruses, are RNA viruses and can be recognized by multiple PRRs, including TLRs, RIG-I-like receptors (RLRs), and NOD-like receptors (NLRs). PRRs can activate their respective signaling pathways to induce the production of IFN-λs and interact with their heterodimeric receptor complexes to activate ISG expression via the JAK/STAT pathway (**Figure 2**).

Of the Toll-like receptors, TLR3/7/8 primarily recognize RNA viruses, with TLR3 sensing double-stranded RNA (dsRNA) and TLR7/8 sensing single-stranded RNA (ssRNA). After recognition of the virus, TLR3 activates the TIR domain-containing adaptor inducing interferon-β (TRIF), whereas TLR7/8 activate myeloid differentiation factor 88 (MyD88). They further activate TANK-binding kinase 1 (TBK1) and the kinase IKK, which induce the production of IFN-λ transcription factors such as IRF3/7 and NF-κB (46, 93, 94). MyD88- and TRIF-dependent pathways result in the phosphorylation and activation of the abovementioned IFN regulators, which ultimately translocate to the nucleus and trigger antiviral immune responses (95, 96). TLR3 is active in antigen-presenting cells and enhances the cross-priming response of dendritic cells (97). The TLR3 agonist Poly (I: C) was found to induce the production of large amounts of IFN-λ, which resulted in the upregulation of cross-reactive costimulatory molecules and enhanced the immune response of cells (97). Another study found that IFN-λ enhanced TLR7-mediated antibody production in human peripheral blood B cells, suggesting that IFN-λ and TLRs do not merely have an upstream or downstream relationship but have a bidirectional regulatory role (89). TLR9 detects the unmethylated CpG DNA of bacteria and viruses, which is the only TLR that detects DNA viruses (98).

RLR is also essential for recognizing RNA viruses, including viruses with either ssRNA or dsRNA. As the primary recognition receptor for cytoplasmic RNA, the RIG-I-MAVS signaling pathway has been extensively investigated in the innate immune system against viral infection. Mitochondrial antiviral signaling protein (MAVS) can activate IRF1/3/7 and NF-κB to induce IFN-λ. This relatively independent IFN-λ induction pathway is not easily targeted by pathogens. MAVS can activate both type I and type III IFNs, but transgenic cell lines preferentially expressing MAVS on peroxisomes only regulate IFN-λ expression by producing IRF1 (99). RIG-I and MDA5 have caspase-activating and recruiting structural domains (CARDs) at their N termini. These CARDs are expressed after an attack by SARS-CoV-2 and activate MAVS-CARD oligomers. RLRs are activated after the recognition of the RNA molecule of the virus, and RLRs and MAVS bind via their respective CARD domains, inducing MAVS to form prion-like structures on mitochondria. Signals are propagated to TBK1 and IKK kinases, which are phosphorylated and activate downstream signaling response (100, 101). NF-κB is activated by MAVS and produces downstream proinflammatory cytokines, recruiting IRF3 transcription factors and ultimately inducing the production of IFN-λ (102, 103).

NLRs can activate cysteine aspartate protease-1 (caspase-1) to produce an inflammatory response similar to that by TLRs. They differ in that NLRs are primarily responsible for the endogenous cellular recognition of viruses in the cytoplasm of affected cells, whereas TLRs are primarily responsible for the exogenous cellular recognition of cells containing internal viruses. NOD-like receptor thermal protein domain-associated protein 3 (NLRP3) is an important member of the NLR family. In the immune system activated by SARS-COV-2, the inflammasome NLRP3 facilitates the triggering of antiviral responses primarily through the production of IFN-λ (104).

### 4.2 Ku70-mediated recognition of DNA viruses

Ku is a heterodimer composed of two subunits of approximately 70 and 80 kDa, referred as Ku70 and Ku80, respectively (105). Ku70, a highly abundant nuclear protein, is a novel DNA sensor that translocates into the cytoplasm upon infection by DNA viruses and induces IFN-λ production instead of IFN-α/β (106). Ku70 activates the downstream TBK1-interferon regulatory factor (IRF) signaling pathway primarily through the STING-dependent signaling pathway to induce the production of IRF-1/3/7, which in turn induce IFN-λ production. In this process, Ku70 colocalizes with Ku80 to form a heterodimeric complex that maintains their respective steady-state levels (107). Nevertheless, Ku80 is not directly involved in the recognition of transduced DNA signals by Ku70 or the activation of downstream signaling pathways. Nuclear localization sequence acetylation is essential for the cytoplasmic localization of Ku70, and its activation of IFN-λ increases in a dose-dependent manner with the accumulation of Ku70 in the cytoplasm. A previous study found that herpes simplex virus, as a typical DNA virus, induces Ku70 nuclear translocation and produces IFN-λ in a virulent strain-dependent manner (108).

### 4.3 STING-mediated recognition of DNA viruses

The stimulator of interferon gene (STING) plays a key role in the downstream pathways of cytoplasmic DNA recognition receptors, which are in an inactivated state and located primarily in the endoplasmic reticulum. Inactive STING acts as a signaling molecule downstream of cyclic GMP-AMP synthase (cGAS), which recognizes cytoplasmic DNA as DAMP and synthesizes cGAMP (109). The critical first step in initiating cGAS-mediated antiviral effects is the synthesis of cGAMP (110). The interaction of cGAS with dsDNA triggers the catalytic activity of cGAS, which allows DNA to be recognized by STING (111, 112). cGAS dimers bind to dsDNA and assemble on it, resulting in the enzymatic activation of cGAS and promotion of cGAMP synthesis. STING located on the endoplasmic reticulum receives upstream signal activation to enhance homodimer formation and translocation to the Golgi complex. It recruits TBK1 and promotes the phosphorylation of TBK1, recruits interferon regulatory factor 3 (IRF3), and activates NF-κB by phosphorylating the kinase IKK (112, 113). These transcription factors are translocated to perinuclear vesicles and promote the production of associated inflammatory cytokines and mRNA expression of type I and type III IFNs, initiating the immune response (109, 114). Recently, cGAS-STING has emerged as a critical driver mediating the pulmonary inflammatory response of COVID-19 (115, 116). The cGAS-STING pathway can promote IFN-λ secretion and STAT1 signal transduction during viral infection (117–119). The function of STING in recognizing DNA makes it play a vital role in recognizing the virus. Ku70 undergoes nuclear translocation, followed by binding to STING in the cytoplasm, and forms a complex that mediates the IFN-λ response to DNA viruses (108). Studies on human macrophages revealed that IFN-λ is a marker of the DNA-mediated STING pathway and that knocking down STING in macrophages inhibits their ability to produce IFN-λ after infection with DNA virus (120).

## 5 Action mechanism of IFN-λ

Type I IFN, type II IFN, and type III IFN all signal through the JAK/STAT pathway that induces ISG transcription, whereas their sites of action and activated STAT complexes are different. IFN-λ has a restricted tissue distribution and acts most prominently in epithelial cells, and IFN-γ is primarily produced by various T and NK cells. They contrast with IFNα/β produced by almost all cell and tissue types. Gene response expression of IFN is driven by STAT dimer, STAT1 homodimer is the major signaling carrier of IFN-γ, and the STAT1–STAT2 heterodimer along with IRF9 forms interferon-stimulated gene factor 3 (ISGF3) to induce the production of type I and type III IFNs (121, 122). IFN-λ and IFNα/β have similar regulatory and biological functions, and both their closely related cytokines possess specific heterodimeric receptors, viz., IFNLR for IFN-λ (IFNLR1 and IL10R2) and IFNAR for IFNα/β (IFNAR1 and IFNAR2). After viral infection, IFN-λ is induced through several similar PRR signaling pathways that induce IFNα/β, but the DNA sensor Ku70 preferentially induces IFN-λ. Transcription factors activated downstream of the signal include IRF1/3/7 and NF-κB. Unlike IRF3 that preferentially binds to IFN-β, IRF7 induces IFN-α to be expressed, IRF1 induces IFN-λ expression, and the combined expression of IRF and NF-κB is essential for the maximal induction of IFN-λ (123). IFN-λ is secreted by infected cells, which then act as a signal to nearby uninfected cells. This paracrine signaling stimulates the production of ISGs, which plays a critical role in inducing an antiviral state through various mechanisms. IFN-λ signals through IFNLR to activate JAK1 and TYK2 kinases, and JAK2 kinase, which exerts no effect on IFN-α/β, may also be activated by IFNLR signaling (123). JAK causes the tyrosine phosphorylation of STAT1 and STAT2 and further forms STAT1-STAT2-IRF9 heterotrimers (ISGF3), which translocate to the nucleus and bind to ISG initiation genes and activate IFN-stimulated regulatory elements (ISREs) to initiate gene transcription and form antiviral effector molecules. Feedback regulation of the IRF1/7-encoded transcription factor IRF1/7 in ISGs produced through this pathway induces the production of IFN-λ (**Figure 2**) (124).

IRFs are a class of transcription factors located in the downstream IFN signaling cascade and are primarily involved in regulating gene expression in response to IFN. All IRF proteins have a conserved amino-terminal DNA-binding domain and a conserved carboxy-terminal IRF-association domain, which are responsible for recognizing ISREs and mediating intramolecular interactions with other transcription factors in homodimers and heterodimers, respectively (125). In the control of gene transcription, IRFs regulate the transcription of different IFNs depending on the species. IRFs bind to the promoter of IFN genes together with NF-κB and activate transcription, where IRF3, IRF7, and NF-κB are required to activate the transcription of type III IFN genes (**Figure 2**). IRF1 is an ISG that is present in all types of cells before viral infection and activates the downstream MAVS pathway; it is only involved in the transcription of the IFN-λ gene and not in the gene induction of type I IFN (123). The expression of both IRF-3 and IRF-7 may be involved in the regulation of IFN-λ1, whereas the induction of IFN-λ2/3 is primarily regulated by IRF-7 (126). NF-κB is involved in the transcription of both type I and type III IFN genes, but type III IFN is more dependent on it. Several specific regulators of IRFs targeting type III IFN have been reported recently. For instance, MED23 directly interacts with IRF7 to synergistically upregulate gene transcription and protein expression levels of IFN-λ (127). RSV inhibits IRF1-induced IFN-λ production through activation of the epidermal growth factor receptor, which further results in lung inflammation (128). IRF9 is a key factor in the JAK/STAT pathway. Phosphorylated STAT1 and STAT2 form an ISGF3 complex with IRF9, which then translocates to the nucleus to advance ISG transcription (96, 117). A cell transfection experiment indicated that STAT1, STAT2, and IRF9 all must be expressed in sufficient levels to induce ISGs, and overexpression of one alone failed to induce ISG when the other two were expressed at very low basal levels. All three components of ISGF3 were required for the induction of ISGs (129).

The induction of ISG through the STAT pathway is generally rapid to respond quickly to viral infection. In humans, type I and type III IFNs can induce antiviral ISGs such as IRF1, IRF7, MX dynamin-like GTPase 1 (MX1), protein kinase R (PKR), interferon-induced protein with tetratricopeptide repeats 1 (IFIT1), and interferon-induced transmembrane protein (IFITM) (130). In IFN-λ-treated mice, 2′-5′-oligoadenylate synthetase 1 (OAS1) and IRF7 were found to be induced in pDCs (131). Although the ISG pools of type I and type III IFNs mostly overlap, there are differences in the kinetics of induction, with type I IFN induction of ISG peaking at an earlier time point and gradually declining and type III IFN induction being more sustained. There are two speculations regarding this situation; one is that the negative regulatory effect of negative regulators such as ISG15 and USP18 on ISG results in the downregulation of type I IFN signaling (132), and the other is that this difference is due to the different intrinsic properties of the two independently expressed signaling pathways (133). These findings require confirmation by further experiments.

## 6 Conclusion

IFN-λ has been extensively investigated, and remarkable achievements have been obtained in the two decades since its discovery in 2003. It plays a vital role in antiviral response and immune regulation with epithelial cell specificity. IFN-λ exerts antiviral effects in cell surface barriers with a higher preference than that of type I IFN. IFN-λ inhibits further progression of infection in epithelial cells early in viral infection, prevents the triggering of type I IFN responses, and inhibits the production of large amounts of inflammatory cytokines to avoid cytokine storms. Nonetheless, recent studies remind us that when using IFN-λ as a treatment strategy, it is necessary to consider the duration of action on the airway barrier and adjust in time according to the severity of the infection.

IFN-λ is associated with the innate immune response mediated by inflammation. IFN-λ recruits neutrophils and NK cells to clear virus-invaded cells and regulates the inflammatory response, acts on DCs to bias T cells toward Th1 antiviral responses, acts directly or indirectly on macrophages to mediate antiviral signaling, and augments antibody production by B cells. IFN-λ is a bridge between intrinsic and adaptive immunity on the surface of the respiratory tract, although the direct activity of IFN-λ on immune cells is limited.

It is important to focus on the differences between immune cells *in vitro* and *in vivo* in IFN-λ studies, as well as further clinical trials are required to address key questions. There also exists a need to deepen biological studies on IFN-λ, consider IFN-λ differences in animals and humans, and design more rigorous and comprehensive study protocols according to the differences.

In recent years, IFN-λ has demonstrated potential for treating respiratory virus-like diseases, especially COVID-19. Its topical antiviral and reduced inflammation-related systemic side effect, considering its tropism for epithelial cells, demonstrates significant therapeutic potential in fighting airway viral infections.

## Author contributions

YL: Writing – original draft, Writing – review & editing. SJ: Writing – review & editing. SZ: Writing – review & editing. TX: Writing – review & editing. YL: Writing – review & editing. RP: Writing – review & editing. MY: Writing – review & editing. QC: Writing – review & editing.
